# An important new health policy voice

**DOI:** 10.1186/s13584-021-00505-6

**Published:** 2021-12-15

**Authors:** Richard B. Saltman

**Affiliations:** Rollins School of Public Health, Emory University, Atlanta, GA USA

**Keywords:** Health systems policy, Health systems analysis, Health system research, Israeli Health System, Comparative Health Systems

## Abstract

In the 10 years since its founding, the Israel Journal of Health Policy Research has established itself as an important voice in Israeli and international health policy. The Journal’s ability to combine national and international perspectives on key issues in health services delivery and health systems analysis has developed a valuable new arena for academic research about the increasingly complex post-COVID future of health care systems.

Journals sit at the very heart of the entire academic enterprise. They are essential not just for individual academics to demonstrate their intellectual acumen, but also, and more importantly, to document and transmit core intellectual knowledge among contemporary and future researchers and practitioners. Successful journals help define our understanding of what is true, and help shape academe’s continually emerging assessment of what further knowledge we require to understand the policy phenomena we confront.

This essential function is particularly important in the complex multi-disciplinary arena that is health policy. Composed of widely varying academic disciplines (economics, political science, sociology, organization theory, management theory along with myriad sub-disciplines), with different, and often divergent, structural environments in different national sometimes within-country regional health delivery arrangements, subject to the complexities of varying national political systems and political cultures, health policy is even more dependent on strong, well-edited, and well-managed journals. Every academic knows the rare pleasure of discovering a smart new journal that publishes important new thinking in the field and that, perhaps, they can publish in themselves.

The arrival of the Israel Journal of Health Policy Research was such an event. The Journal has grown in breadth, depth, and, consequently, stature throughout its 10 year history, resulting in what is now an important international as well as national voice in the increasingly complex COVID-driven policy environment that developed country health systems now confront. Faced with the necessity of re-assessing and re-evaluating their core regulatory and operational arrangements, countries from Sweden [1] to the United Kingdom [2, 3] and Spain [4], as well as the European Union in conjunction with the WHO Regional Office for Europe through its high level commission on health system resilience [5, 6], are seeking better answers and better strategies for dealing with future public health crises. It is in part to the credit of the Israel Journal of Health Policy Research that Israeli policymaking before and during the COVID-19 epidemic, especially around population vaccination strategies, rates, and outcomes, has played an important international role in this ongoing international debate [7].

Academic journals are not easy enterprises to develop, however. They are notoriously difficult to start, to staff, and to steer in an effective and successful manner. That developmental process takes enormous organizational, intellectual, and sometimes physical fortitude, much of it long before the outcome can be known. The Israel Journal wisely positioned itself strategically astride both the rapidly developing Israeli health policy world, and the broader policy debates across Europe and North America.

Initial international discussions about the proposed journal’s focus were held at a meeting of the International Advisory Committee of the Israel National Health Policy Institute, held during its widely attended Global Conference on Health Policy. Suggestions from senior academics from organizations like the European Observatory on Health Systems and Policies, the World Bank, University College London, London School of Hygiene and Tropical Medicine, and other major research institutions in Europe and the United States helped shape the international component of the new journal. Many of these individuals later contributed commentaries or articles as the Journal took form, strengthening its scope and reach, and confirming the wisdom of the founding editors’ combined domestic and international vision.

It is to the great credit of the Journal’s editors and sponsors that it has come so far so fast. Those of us in the international community who have worked with them look forward to continuing this wonderful interaction in the coming years.

## Data Availability

Not applicable.
